# Multiterritory Bihemispheric Ischemic Stroke in the Setting of Unilateral Internal Carotid Artery Disease

**DOI:** 10.7759/cureus.115353

**Published:** 2026-08-28

**Authors:** Kalina Misiolek, Amna Sohail

**Affiliations:** 1 Neurology, Cleveland Clinic Foundation, Cleveland, USA; 2 Neurology, University of Virginia, Charlottesville, USA

**Keywords:** anterior cerebral, anterior cerebral artery (aca), artery-to-artery embolism, bi-hemispheric infarction, carotid artery stenosis, circle of willis variant, ischemic stroke, ischemic stroke mechanism

## Abstract

Accurate determination of stroke mechanism is essential for secondary stroke prevention. Bilateral, multiterritory ischemic infarcts are commonly attributed to a central embolic source, prompting evaluation for cardioembolism, paradoxical embolism, or hypercoagulable states. However, this diagnostic approach assumes conventional intracranial vascular anatomy despite the high prevalence of Circle of Willis variants. We report the case of a 66-year-old man who presented with acute bilateral cortical and subcortical ischemic infarcts involving the right middle cerebral artery (MCA), right anterior cerebral artery (ACA), and left ACA territories. Although transthoracic echocardiography identified a patent foramen ovale (PFO), digital subtraction angiography (DSA) demonstrated a hypoplastic left A1 segment with the left ACA supplied by the right internal carotid artery (ICA) through a patent anterior communicating artery (ACom). This vascular configuration provided a plausible pathway by which artery-to-artery emboli arising from the ulcerated right proximal cervical ICA plaque could reach both hemispheres. The collective clinical and imaging findings supported the right ICA plaque as the most likely culprit source, prompting carotid endarterectomy. This case highlights the importance of incorporating individualized cerebrovascular anatomy into stroke mechanism determination and secondary stroke prevention.

## Introduction

Stroke remains a leading cause of death and long-term disability worldwide, and accurate determination of stroke mechanism is fundamental to selecting appropriate secondary prevention strategies [1]. Multiterritory ischemic stroke often prompts evaluation for a proximal central embolic source, including atrial fibrillation, other cardioembolic conditions, and hypercoagulable states, because simultaneous infarction in multiple vascular territories is commonly attributed to these mechanisms [2,3]. However, this diagnostic approach assumes conventional intracranial vascular anatomy.

Anatomical variants of the Circle of Willis are common, with reported prevalence estimates of approximately 50%-60%, although estimates vary according to study population, imaging modality, and definitions used [4,5]. In conventional anterior circulation anatomy, each internal carotid artery (ICA) supplies the ipsilateral anterior cerebral artery (ACA) through its A1 segment, while the two ACA circulations are connected by the anterior communicating artery (ACom) [5]. Variants involving this anatomy may substantially alter collateral flow and potential embolic pathways [5]. When one A1 segment is markedly hypoplastic and the ACom is patent, the contralateral ICA may provide dominant blood supply to both ACA territories, providing a potential anatomical pathway linking unilateral carotid disease with infarcts in otherwise unexpected vascular distributions [5].

Previous reports have described contralateral or bilateral ACA infarction in the setting of unilateral carotid disease and anterior circulation variants [6,7]. The present case extends these observations by demonstrating simultaneous right middle cerebral artery (MCA) and bilateral ACA infarction in the setting of unilateral right cervical ICA disease, with digital subtraction angiography (DSA) demonstrating right-to-left ACA cross-filling. This case highlights how consideration of individualized cerebrovascular anatomy may help explain an apparently discordant multiterritory infarct pattern and inform assessment of a potential unilateral large-artery source.

## Case presentation

A 66-year-old right-handed man with hypertension and hyperlipidemia presented to another institution in December 2022 with a left MCA territory transient ischemic attack (TIA), manifested by right arm and hand weakness, in the setting of severe left cervical ICA atherosclerotic stenosis demonstrated on computed tomography angiography (CTA) of the head and neck, for which he underwent left carotid endarterectomy (CEA). The CTA at that time also demonstrated asymptomatic 48% stenosis of the right proximal cervical ICA. His home medications included aspirin 81 mg daily, rosuvastatin 10 mg daily, and tamsulosin 0.4 mg daily; hypertension was managed with lifestyle measures. In January 2023, he presented to an outside hospital (OSH) with sudden-onset left hemiparesis, greater in the leg than the arm and face, left hemisensory loss, right leg weakness, and mild dysarthria. He was evaluated by tele-neurology at the OSH, with an initial National Institutes of Health Stroke Scale (NIHSS) [8] score of 13: left arm weakness (3), left leg weakness (4), right leg weakness (3), sensory loss (1), dysarthria (1), and facial weakness (1). Blood pressure was 152/67. Presenting laboratory evaluation at the OSH demonstrated a serum glucose of 130 mg/dL, with renal function, coagulation parameters, platelets, and troponin I within normal limits.

Intravenous tenecteplase (TNK; 0.25 mg/kg, 19.6 mg total dose) was administered as a single bolus at the OSH exactly three hours after symptom onset, resulting in marked neurological improvement. Following transfer to our institution, his NIHSS score had improved to 2, with residual left leg drift (1 point) and left-sided sensory loss (1 point). At that time, he described his left leg as feeling as though it was “not his own” and reported a loss of control over the limb. This disturbance in the sense of limb ownership and voluntary control was clinically characterized as an alien limb phenomenon.

**Figure 1:**
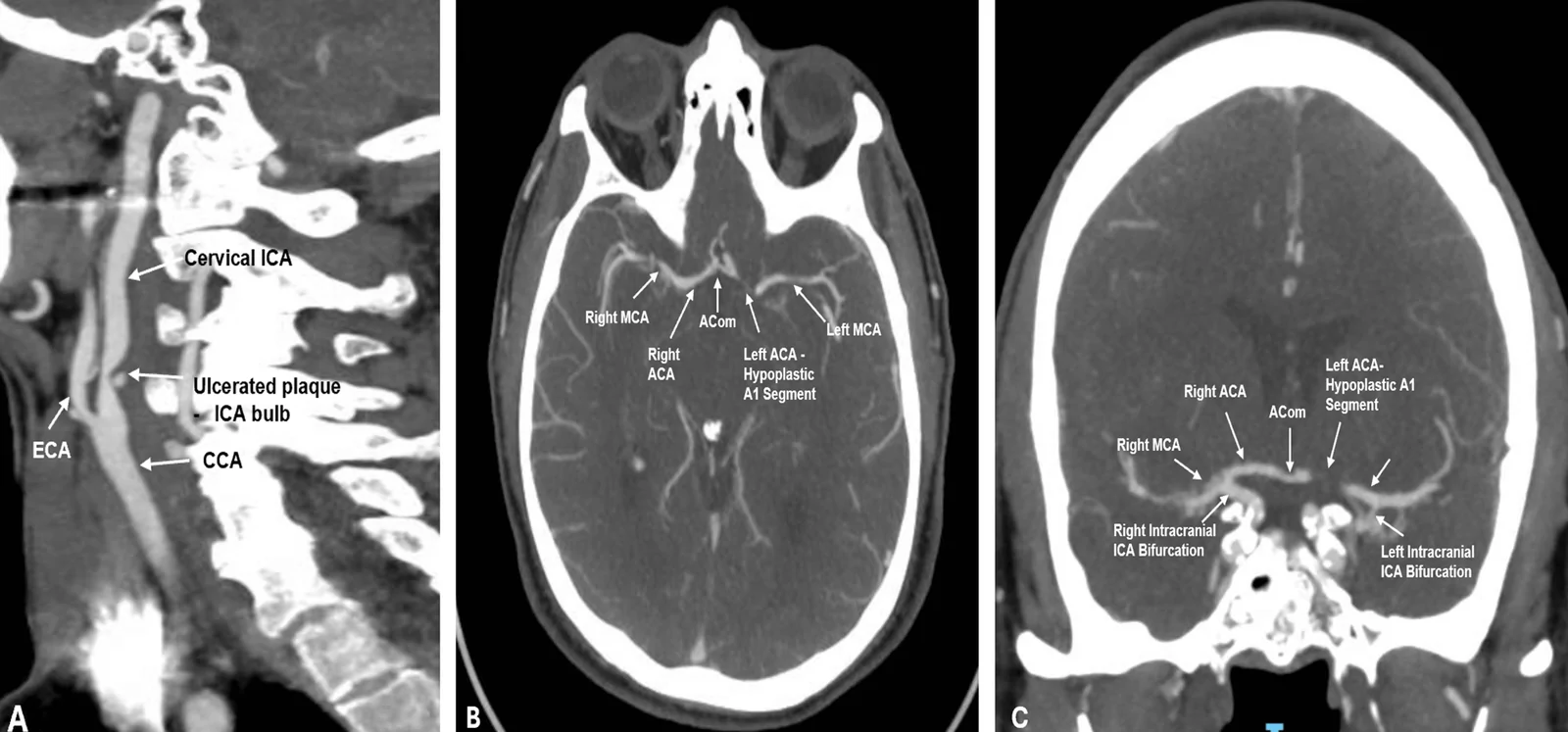
Computed Tomography Angiography Demonstrating Right Cervical Internal Carotid Artery Stenosis With Plaque Ulceration and a Hypoplastic Left A1 Segment. (A) Sagittal CT angiography of the neck demonstrating an ulcerated plaque involving the right proximal cervical internal carotid artery (ICA), resulting in 72% stenosis by North American Symptomatic Carotid Endarterectomy Trial (NASCET) criteria, with a minimal residual luminal diameter of 1.2 mm and a distal reference ICA diameter of 4.3 mm. (B) Axial CT angiography of the head demonstrating marked asymmetry of the A1 segments and a hypoplastic left A1 segment. (C) Coronal CT angiography, on which the A1 segments were best visualized, demonstrating a left A1 diameter of 0.87 mm compared with 2.40 mm on the right. ACA, anterior cerebral artery; ACom, anterior communicating artery; CCA, common carotid artery; ECA, external carotid artery; ICA, internal carotid artery; MCA, middle cerebral artery.

Following transfer, CTA of the head and neck performed at our institution demonstrated no intracranial large-vessel occlusion; therefore, mechanical thrombectomy was not indicated. The same study demonstrated 72% stenosis of the right proximal cervical ICA by North American Symptomatic Carotid Endarterectomy Trial (NASCET) criteria [9], with an irregular ulcerated plaque characterized by focal contrast extension beyond the expected luminal boundary into the plaque (Figure 1A). Comparison by our neuro-radiologists with the CTA images obtained one month earlier, in which the right proximal cervical ICA stenosis measured 48%, demonstrated interval progression to 72%. Both examinations were assessed using a comparable NASCET methodology, while acknowledging potential interscan measurement variability. The irregular intraluminal component associated with the ulcerated plaque raised concern for superimposed thrombus, which could not be reliably excluded on CTA. Magnetic resonance angiography (MRA) of the neck was therefore obtained to further evaluate the irregular intraluminal component because confirmation of superimposed thrombus would have affected antithrombotic management. MRA demonstrated severe right proximal cervical ICA stenosis with plaque ulceration but no evidence of superimposed thrombus. The institutional MRA protocol was not designed to evaluate intraplaque hemorrhage. 

**Figure 2:**
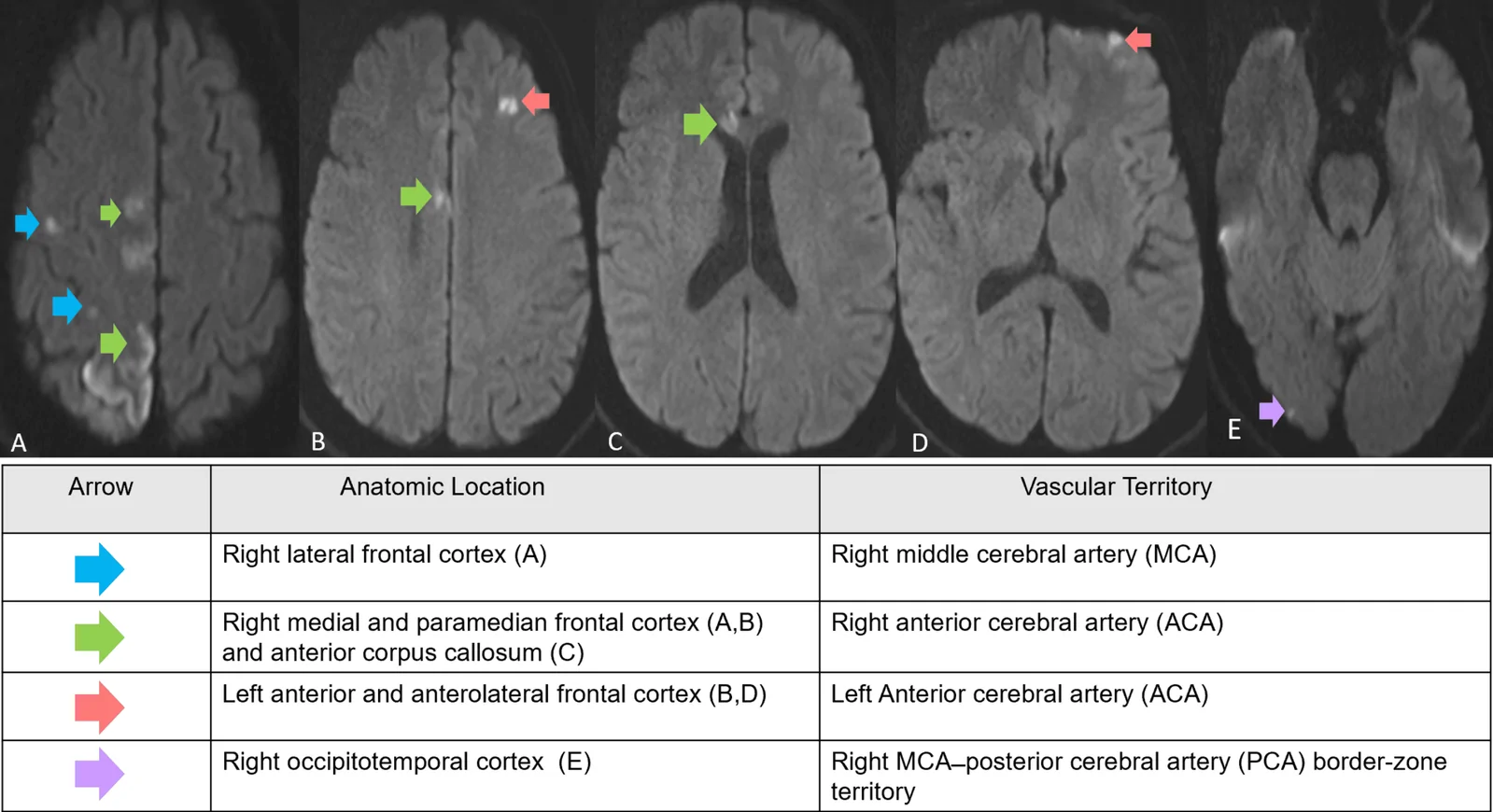
Diffusion-Weighted Magnetic Resonance Imaging Demonstrating Multiterritory Acute Ischemic Infarcts. Diffusion-weighted magnetic resonance imaging demonstrating multiple punctate acute cortical and subcortical infarcts involving the right middle cerebral artery (MCA), right anterior cerebral artery (ACA), and left ACA distributions. Blue arrows indicate right lateral frontal cortical infarcts in the right MCA territory; green arrows indicate right medial/paramedian frontal and anterior corpus callosal infarcts in the right ACA territory; red arrows indicate left frontal infarcts in the distal left ACA distribution, including the ACA-MCA cortical border-zone region; and the purple arrow indicates a right MCA-posterior cerebral artery (PCA) cortical border-zone infarct.

Brain magnetic resonance imaging (MRI) demonstrated multiple punctate acute cortical and subcortical infarcts involving the right MCA and right ACA territories, the distal left ACA distribution including the ACA-MCA cortical border-zone region, and the right MCA-PCA cortical border-zone region (Figure 2). Infarcts involving the anterior corpus callosum and adjacent ACA territory provided a plausible anatomical correlate for the patient's alien limb phenomenon. The bilateral, multiterritory infarct distribution initially raised concern for a central embolic source.

Additional laboratory evaluation demonstrated marked dyslipidemia, with a low-density lipoprotein cholesterol level of 175 mg/dL, total cholesterol of 304 mg/dL, and triglycerides of 387 mg/dL; hemoglobin A1c was 5.6%. The patient was a nonsmoker.

Transthoracic echocardiography (TTE) with agitated saline demonstrated evidence of a patent foramen ovale (PFO). Given the multiterritory infarct pattern, the echocardiographic findings were reviewed with cardiology, who confirmed the absence of spontaneous right-to-left shunting at rest, atrial septal aneurysm, or other high-risk PFO features. Cardiology considered the TTE image quality adequate and did not recommend additional evaluation with transesophageal echocardiography (TEE). The Risk of Paradoxical Embolism (RoPE) score was 4, indicating a lower probability that the PFO was related to the stroke. Bilateral upper- and lower-extremity venous duplex ultrasonography demonstrated no evidence of deep venous thrombosis to support paradoxical embolism through the PFO. The initial electrocardiogram (EKG) and continuous inpatient telemetry during the first four days of hospitalization detected no atrial fibrillation, atrial flutter, or other sustained arrhythmia. The patient had no clinical features suggestive of occult malignancy and was up to date with age-appropriate cancer screening.

**Figure 3:**
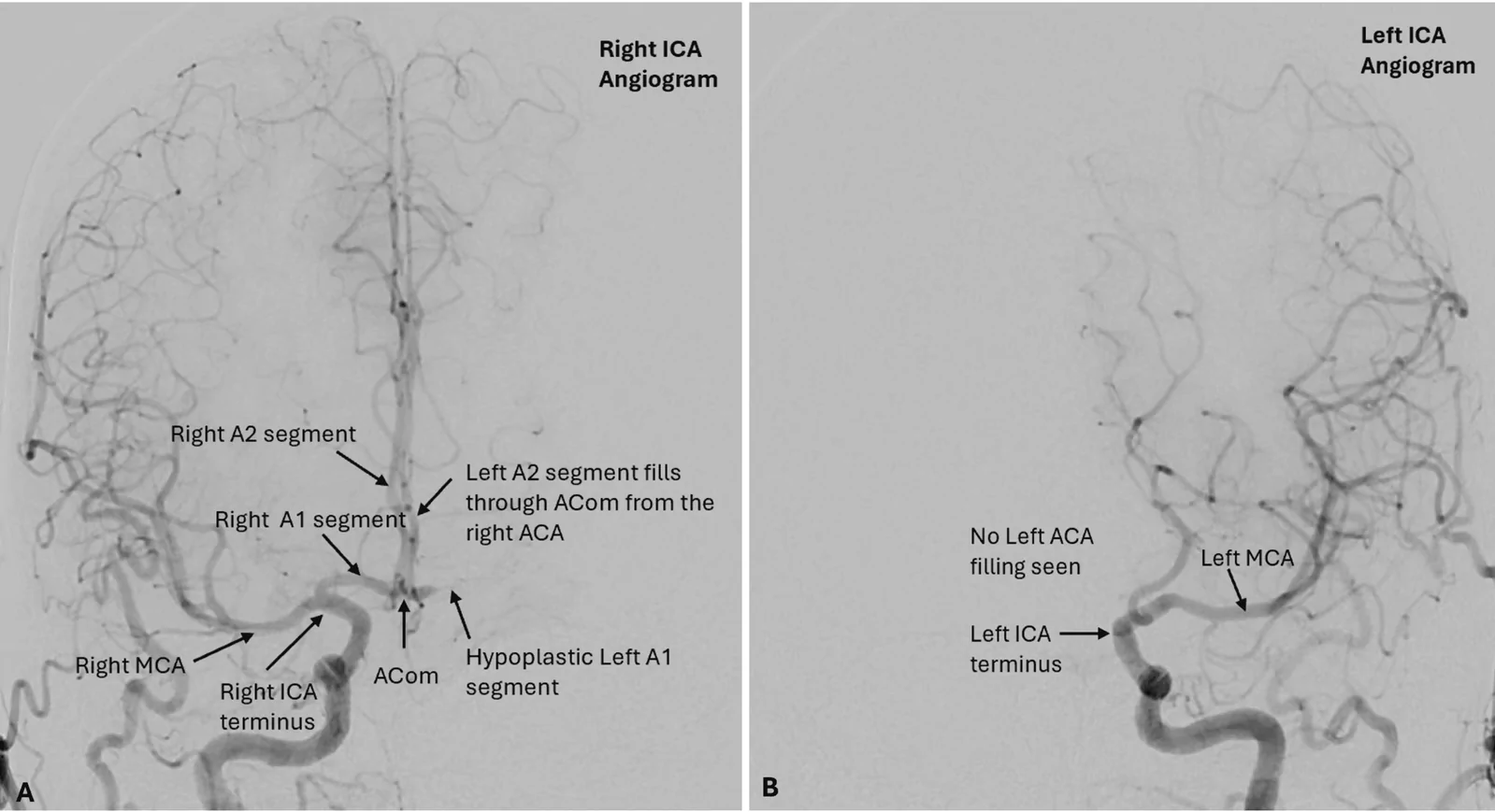
Digital Subtraction Angiography Demonstrating Cross-Filling of the Left Anterior Cerebral Artery Through the Anterior Communicating Artery. (A) Digital subtraction angiography following right internal carotid artery (ICA) injection in an anteroposterior (AP) projection during the arterial phase, demonstrating opacification of both anterior cerebral artery (ACA) territories, including cross-filling of the left ACA through the anterior communicating artery (ACom), with a markedly hypoplastic left A1 segment. (B) Digital subtraction angiography following left ICA injection in an AP projection during the arterial phase, demonstrating absent opacification of the left ACA, confirming functional supply of the left ACA territory from the right ICA through the ACom.

Careful review of the CTA demonstrated marked asymmetry of the A1 segments, best visualized on coronal imaging, with the left A1 measuring 0.87 mm in diameter compared with 2.40 mm on the right. A patent ACom was also identified (Figures 1B, 1C). Although CTA demonstrated the anatomical configuration, it could not establish the functional source of blood flow to the left ACA territory. DSA was therefore performed to further characterize the anterior circulation and determine whether the right ICA functionally supplied the left ACA territory through the ACom, thereby assessing whether the right ICA represented a potential large-artery source for the observed infarct distribution. Right ICA injection demonstrated opacification of both ACA territories through the ACom, whereas left ICA injection demonstrated no opacification of the left ACA, confirming functional supply of the left ACA territory from the right ICA through the ACom (Figure 3). DSA also demonstrated approximately 60% right cervical ICA stenosis.

Carotid duplex ultrasonography was subsequently performed to provide additional characterization of the right ICA plaque morphology and echogenicity, including features associated with increased embolic potential, such as surface ulceration and hypoechoic plaque composition. Duplex also provided complementary hemodynamic assessment through Doppler velocity measurements, which was particularly relevant given the moderate degree of stenosis demonstrated on DSA and the cortical border-zone distribution of some of the infarcts. Duplex demonstrated an ulcerated, hypoechoic plaque within the proximal right cervical ICA, with recorded velocities of 191 cm/s in the proximal ICA, 132 cm/s in the common carotid artery (CCA), and 331 cm/s in the external carotid artery (ECA). Collectively, the vulnerable right ICA plaque, demonstrated right-to-left ACA cross-filling, and absence of a compelling alternative embolic source supported the right ICA plaque as the most likely culprit source.

On the basis of the overall clinical and imaging findings, secondary stroke prevention included aspirin 81 mg daily and escalation to high-intensity statin therapy with rosuvastatin 40 mg daily. Right CEA was subsequently performed on post-stroke day 7. The operative report described a ruptured hemorrhagic atheroma encountered during endarterectomy; however, no histopathology report or histopathologic images from the right carotid endarterectomy were available. The postoperative course was uncomplicated, and the alien limb phenomenon resolved before discharge. Inpatient telemetry remained without atrial fibrillation, atrial flutter, or other sustained arrhythmia throughout the remainder of the hospitalization. A subsequent seven-day ambulatory cardiac monitor likewise detected no atrial fibrillation or atrial flutter. At three-month follow-up, he had returned to work with minimal residual deficits. At three years, he remained free of recurrent stroke or TIA, with an NIHSS score of 1 for residual left leg sensory loss and a modified Rankin Scale (mRS) [10] score of 1.

## Discussion

The Circle of Willis is the principal collateral network of the cerebral circulation, and anatomical variants can substantially alter both cerebral blood flow and expected embolic pathways [5]. Hypoplasia of the A1 segment of the ACA has been reported in approximately 10%-27% of individuals, whereas complete A1 aplasia occurs in approximately 1%-9% [11-13]. Definitions of A1 hypoplasia vary across studies, with commonly used criteria including an A1 diameter <1 mm [11] or <50% of the contralateral A1 diameter [14]. In our patient, the left A1 measured 0.87 mm compared with 2.40 mm on the right on coronal CTA, representing approximately 36% of the contralateral A1 diameter and satisfying both criteria for A1 hypoplasia. When one A1 segment is hypoplastic and the ACom is patent, the contralateral ICA may provide dominant blood supply to both ACA territories, altering both collateral perfusion and potential embolic pathways [15]. The functional significance of this anatomy was demonstrated on DSA: left ICA injection did not opacify the left ACA, whereas right ICA injection opacified both ACA territories through the ACom. Thus, the left A1 was morphologically hypoplastic, with the left ACA territory functionally supplied by the right ICA. Other anterior circulation variants, including azygos, bihemispheric, and accessory ACAs, can similarly alter expected collateral and embolic pathways (Table 1) [11-13,16].

**Table 1:** Common Anterior Cerebral Artery Variants and Potential Ischemic Stroke Implications. Prevalence estimates vary substantially according to study population, anatomical definition, and methodology, including cadaveric, angiographic, computed tomography angiography (CTA), and magnetic resonance angiography (MRA) studies; representative estimates and corresponding references are therefore provided for each variant. Expected collateral circulation and potential ischemic stroke implications are synthesized from anatomical studies [11-13,16] and hemodynamic data regarding A1 asymmetry [15].

Variant	Approximate Prevalence	Expected Collateral Circulation	Potential Ischemic Stroke Implication
A1 hypoplasia	10-27% [11,12]	Increased dependence on the contralateral ICA through a patent ACom	Unilateral carotid disease may produce contralateral ACA infarction
A1 aplasia	~1-9% [11-13]	Complete dependence on the contralateral ICA for ACA perfusion	Greater potential for contralateral embolization into the opposite ACA territory
Azygos ACA	~0.3-2% [12,16]	A single A2 trunk supplies both medial frontal hemispheres	Single-vessel occlusion may produce bihemispheric ACA infarction
Bihemispheric ACA	Variable; uncommon [12,16]	One ACA provides dominant supply to both hemispheres because the contralateral ACA is hypoplastic or absent	Unilateral embolization may produce bihemispheric ACA infarction
Accessory ACA (persistent median artery of the corpus callosum)	Variable; reported up to ~13% [11,16]	An additional ACA arising from the ACom contributes to medial frontal and callosal perfusion	May alter collateral circulation and expected infarct distribution

Growing evidence suggests that Circle of Willis variants are not merely incidental anatomical findings but can meaningfully influence stroke localization and mechanism. Rangus et al. demonstrated that assessment of Circle of Willis anatomy resulted in reclassification of stroke mechanism in 8.4% of patients with multiterritory infarction, highlighting the importance of collateral anatomy when interpreting infarct distribution [17]. Similarly, Merino and Tavarez emphasized that cerebrovascular variants may alter expected vascular territories and should be considered when the observed infarct pattern appears discordant with the presumed culprit vessel [5]. These observations support incorporating individualized cerebrovascular anatomy into stroke mechanism determination, particularly when conventional vascular territories do not adequately explain the distribution of ischemic injury.

**Figure 4:**
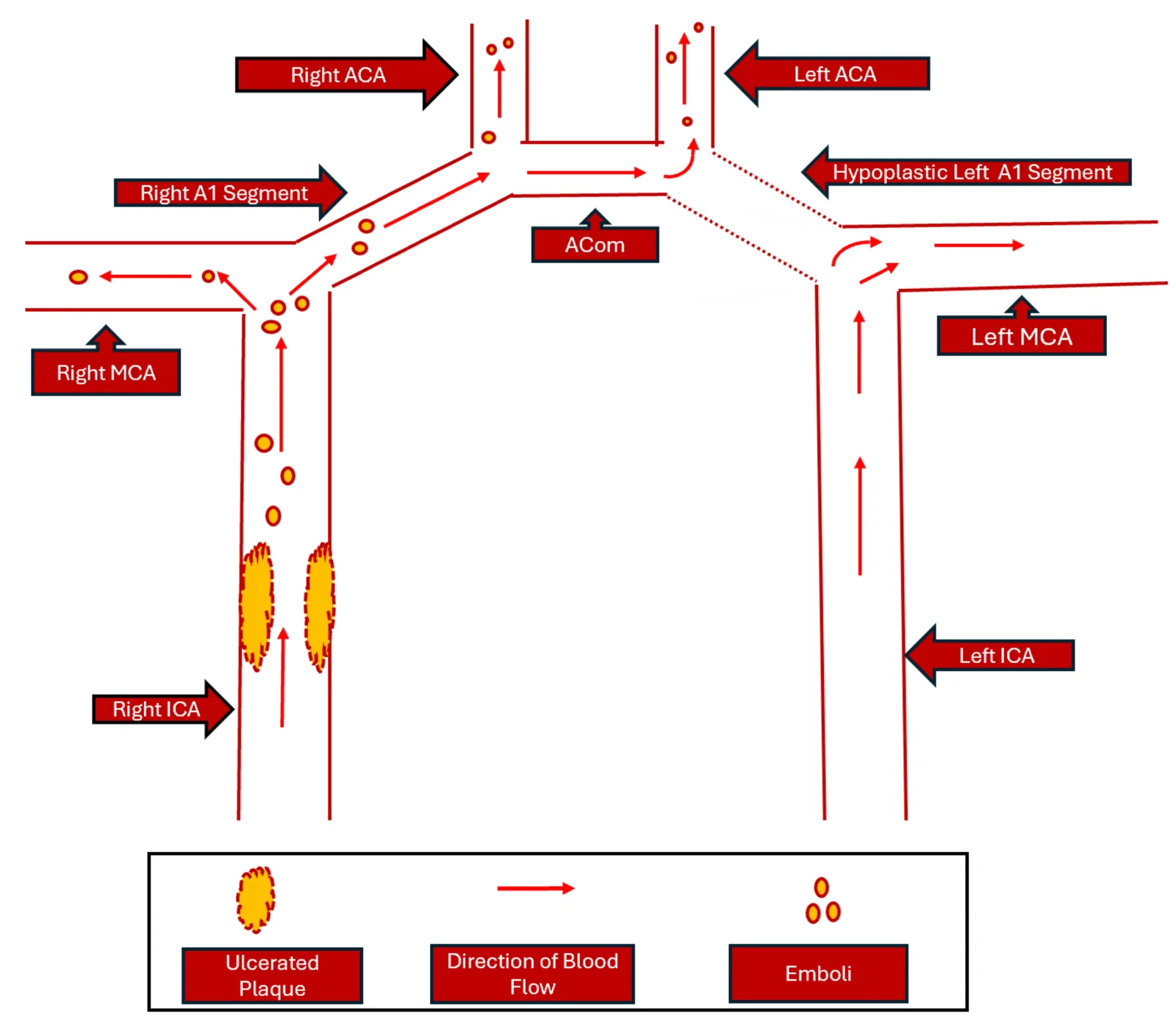
Proposed Embolic Mechanism in the Setting of a Hypoplastic Left A1 Segment. Schematic illustrating the proposed artery-to-artery embolic mechanism from an ulcerated right cervical internal carotid artery (ICA) plaque. In the presence of a hypoplastic left A1 segment of the anterior cerebral artery (ACA) and a patent anterior communicating artery (ACom), the left ACA territory is supplied predominantly through the right ICA. This configuration could permit emboli arising from the right ICA plaque to enter the ipsilateral right MCA and ACA territories and traverse the ACom to reach the contralateral left ACA territory.

In the present case, simultaneous right MCA and bilateral ACA infarction occurred in the setting of unilateral right cervical ICA disease. DSA demonstrated that the right ICA functionally supplied both ACA territories through the ACom, establishing an anatomical pathway through which emboli originating within the right carotid circulation could potentially reach the ipsilateral MCA and ACA territories as well as the contralateral ACA territory. This configuration offers a plausible anatomical explanation for an infarct pattern that initially suggested a proximal central embolic source (Figure 4).

Cortical border-zone infarcts may arise through embolic or hemodynamic mechanisms, and the two mechanisms may coexist. External or cortical border-zone infarcts have been associated predominantly with embolic mechanisms, with or without concomitant hypoperfusion, whereas internal border-zone infarcts are more strongly associated with hemodynamic compromise [18]. In the present case, the distal left ACA-MCA and right MCA-posterior cerebral artery (PCA) lesions involved cortical border-zone regions, with no internal border-zone infarcts identified; the remaining multifocal infarcts also demonstrated a distribution more suggestive of embolization. A hemodynamic or mixed embolic-hemodynamic contribution was nevertheless considered given the cortical border-zone involvement. DSA demonstrated approximately 60% right ICA stenosis, with carotid duplex showing no hemodynamic evidence of critical flow limitation. Transient hypoperfusion preceding these studies cannot be excluded and may have contributed to the cortical border-zone infarcts.

Several findings supported the right ICA plaque as the most likely culprit source for his stroke. The infarcts involved multiple territories functionally supplied by the right ICA, including the contralateral ACA territory as demonstrated by DSA. The right ICA plaque demonstrated ulceration and hypoechoic morphology on vascular imaging, with a ruptured hemorrhagic atheroma subsequently described at endarterectomy. Alternative embolic sources were also evaluated. The PFO lacked high-risk features and was not accompanied by evidence of deep venous thrombosis, while inpatient and ambulatory cardiac monitoring did not identify atrial fibrillation or atrial flutter. No atrial fibrillation or atrial flutter was documented in the available medical record during three years of subsequent clinical follow-up, although intermittent paroxysmal atrial fibrillation cannot be definitively excluded.

## Conclusions

This case highlights the importance of integrating individual cerebrovascular anatomy into stroke mechanism determination. Anatomical variants of the Circle of Willis may provide a pathway linking unilateral carotid artery disease with unexpected bihemispheric, multiterritory infarct patterns that mimic a proximal central embolic source. Recognition of these variants can help reconcile an otherwise discordant infarct distribution and inform mechanism-directed secondary stroke prevention.
